# Candidate Effector Pst_8713 Impairs the Plant Immunity and Contributes to Virulence of *Puccinia striiformis* f. sp. *tritici*

**DOI:** 10.3389/fpls.2018.01294

**Published:** 2018-09-11

**Authors:** Mengxin Zhao, Jianfeng Wang, Sen Ji, Zengju Chen, Jinghua Xu, Chunlei Tang, Shuntao Chen, Zhensheng Kang, Xiaojie Wang

**Affiliations:** State Key Laboratory of Crop Stress Biology for Arid Areas, College of Plant Protection, Northwest A&F University, Yangling, China

**Keywords:** effector, virulence, stripe rust, biotrophic, wheat (*Triticum aestivum*)

## Abstract

*Puccinia striiformis* f. sp. *tritici* (*Pst*), the causal agent of stripe rust, is an obligate biotrophic pathogen responsible for severe wheat disease epidemics worldwide. *Pst* and other rust fungi are acknowledged to deliver many effector proteins to the host, but little is known about the effectors’ functions. Here, we report a candidate effector Pst_8713 isolated based on the genome data of CY32 and the expression of *Pst_8713* is highly induced during the early infection stage. The *Pst_8713* gene shows a low level of intra-species polymorphism. It has a functional N-terminal signal peptide and its product was found in the host cytoplasm and nucleus. Co-infiltrations in *Nicotiana benthamiana* demonsrated that Pst_8713 was capable of suppressing cell death triggered by mouse pro-apoptotic protein-BAX or *Phytophthora infestans* PAMP-INF1. Overexpression of Pst_8713 in plants suppressed pattern-triggered immunity (PTI) -associated callose deposition and expression of PTI-associated marker genes and promoted bacterial growth *in planta*. Effector-triggered immunity (ETI) induced by an avirulent *Pst* isolate was weakened when we overexpressed Pst_8713 in wheat leaves which accompanied by reduction of reactive oxygen species (ROS) accumulation and hypersensitive response (HR). In addition, the host induced gene silencing (HIGS) experiment showed that knockdown of *Pst_8713* weakened the virulence of *Pst* by producing fewer uredinia. These results indicated that candidate effector Pst_8713 is involved in plant defense suppression and contributes to enhancing the *Pst* virulence.

## Introduction

Plant innate immunity provides a defense against pathogens and attacks from parasites (Jones and Takemoto, 2004). It is now understood that the plant immune system generally involves two layers of defense. The first layer involves the recognition of pathogen associated molecular patterns (PAMPs), such as chitin in fungi or flagellin in bacteria. PAMPs are recognized by plant transmembrane pattern recognition receptors (PRRs) and trigger PAMP-triggered immunity (PTI) including callose deposition and expression of defense related genes (Chisholm et al., 2006; Jones and Dangl, 2006). Successful pathogens deploy effectors to promote virulence and compatibility (Jones and Dangl, 2006; Sohn et al., 2007). Effectors can interfere with PTI and force plants into the next layer of immune defense system (Jones and Dangl, 2006). However, many effectors are recognized by plant NB-LRR proteins inside the plant cells, resulting in effector-triggered immunity (ETI), which usually comes with localized hypersensitive response (HR) and Reactive Oxygen Species (ROS) bursts (Jones and Dangl, 2006). Many effectors were assumed to interfere with plant immunity (PTI or ETI) by creating an in-host environment conducive for pathogens to grow and reproduce (Sohn et al., 2007; Guo et al., 2009; Wang et al., 2011; Dou and Zhou, 2012).

Wheat stripe rust, caused by basidiomycetous fungus *Puccinia striiformis* f. sp. *tritici* (*Pst*), is a prevalent and destructive disease of wheat in most wheat growing areas (Wellings, 2011; Chen et al., 2014). In the 21st century, wheat stripe rust has become one of the biggest biotic threats to global wheat production (Schwessinger, 2017). Thus, an understanding of the molecular basis of *Pst* pathogenesis and *Pst*–wheat interaction should enable us to develop new strategies to sustainably control stripe rust. Unlike many necrotrophic fungi, *Pst* is an obligate biotrophic parasite that is not culturable *in vitro* (Staples, 2000). The pathogen can only infect live host tissues, in which it forms haustoria that grow into host cells to uptake nutrients (Voegele et al., 2009; Voegele and Mendgen, 2011). Meanwhile, a large number of effectors are secreted by haustoria to interfere with host defense (Catanzariti et al., 2007).

The use of the next generation sequencing technology has dramatically increased the number of sequences of fungal pathogen genomes and transcriptomes, which has enabled the identification of effectors in many fungi, including *Pst* (Mosquera et al., 2009; Cabral et al., 2011; Cantu et al., 2013; Xia et al., 2017). Accordingly, the role of pathogen effectors has become an important topic in the study of molecular plant pathology, but less is known about rust effectors (Duplessis et al., 2011). Cantu et al. (2013) identified five *Pst* candidate effectors which were haustorial expressed secreted proteins with polymorphism by resequencing genomes from four US and UK *Pst* isolates. Because *Pst* lacks a stable and efficient transformation system, few *Pst* effectors have been studied at the function level (Petre et al., 2014). The host-induced gene silencing (HIGS) technique has greatly facilitated research on pathogenicity progressing and has enhanced the study of *Pst* effectors (Nowara et al., 2010; Yin and Hulbert, 2015). Meanwhile, Yin and Hulbert (2011) reported the use of bacterial type three secretion systems (TTSS) to deliver proteins into wheat cells, which is feasible for studying the functions of *Pst* effectors. It is expected that more research will be conducted on *Pst* effectors, which will lead to a better understanding of effector function in *Pst*–wheat interaction.

In this study, we isolated and characterized a candidate effector gene, designated as *Pst_8713* which was highly induced in *Pst*-infected wheat leaves. By overexpressing Pst_8713 in plants and silencing the *Pst_8713* gene with BSMV-mediated HIGS, we were able to investigate the function of Pst_8713. Our results showed that Pst_8713 significantly impairs the plant immunity and plays an important role in rust pathogenicity.

## Materials and Methods

### Plant Materials, Fungal Isolates, and Bacterial Strains

Wheat cultivars Suwon11 and AvS, isolates of *Pst* CYR23 (avirulent on Su11) and CYR32 (virulent on Su11), and tobacco (*Nicotiana benthamiana*) were used in this study. Wheat and *N. benthamiana* plants were grown at 16 and 23°C, respectively. The second leaves of wheat seedlings at the two-leaf stage were collected for wheat mesophyll protoplast preparation. The isolates of CYR32 and CYR23 were routinely grown and maintained on wheat cultivars Suwon11 and Mingxian169, respectively (Kang et al., 2002). Fresh urediniospores of CYR32 were collected and incubated in water at 9–10°C in dark for 10 h, to allow for germination and germ-tube collection. *Agrobacterium tumefaciens* strain GV3101 was used for transient expression in *N. benthamiana*. *Pseudomonas fluorescens* strain EtHAn was used for TTSS. Yeast isolate YTK12 was cultured in YPDA liquid medium at 30°C.

### Plasmid Construction and Preparation

Primers used for plasmid construction are given in Supporting Information (**Supplementary Table S1**). Primers were designed based on the *Pst_8713* cDNA sequence containing the complete ORF from the genome data of wheat (cv. Suwon11) leaves infected with *Pst* isolate CYR32. *Pst_8713* was cloned from the cDNA of *Pst* isolate CYR32 using FastPfu DNA polymerase (TransGen Biotech, Beijing, China). To validate the secretion function, the predicted signal peptide sequences of Pst_8713 and the oomycete effector Avr1b, as well as the first 25 amino acids of the Mg87 protein from *Magnaporthe oryzae* were fused to the vector pSUC2. To confirm the subcellular localization of Pst_8713 in wheat protoplasts and *N. benthamiana*, its open reading frame (ORF) sequence without the signal peptide was cloned into the pTF486 (Yu et al., 2008) and pK7WGF2 vectors (Karimi et al., 2002). To silence *Pst_8713*, a 187 bp fragment containing a part of the 97 bp untranslated region with a part of the coding sequence, along with a 184 bp fragment containing the part of the coding sequence with the 67 bp untranslated region, were cloned into the BSMV gamma vector (Holzberg et al., 2002). The fragments did not show significant homology with any other *Pst* or wheat genes by BLAST analyses, indicating their specificity. To overexpress Pst_8713 in *N. benthamiana* and wheat, its sequence encoding mature proteins without the putative signal peptide of *Pst_8713* was cloned into the PVX vector pGR107 (Jones and Baulcombe, 1999) and pEDV6 (Sohn et al., 2007).

### Domain Prediction, Sequence Alignment, and Polymorphism Analysis

To check whether *Pst_8713* has any homologs in other organisms, the amino acid sequence of Pst_8713 was analyzed using BLASTP and compared with non-redundant databases (NCBI) (Stephen et al., 1997). SignalP 4.1^1^ was used to predict the signal peptide of Pst_8713.

To determine the polymorphism of Pst_8713, we compared the amino acid sequences of 11 *Pst* isolates from China (CYR32), the US (PST-21, PST-43, PST-78, and PST-130), the UK (PST08/21 and PST87/7) and India (Races 31, K, Yr9, and 38S102). The genomes of CYR32 (Zheng et al., 2013), PST-78, re-sequenced genomes of PST-21, PST-43, PST-130, PST08/21, and PST87/7 (Cantu et al., 2013) and 4 Indian isolates (Races 31, K, Yr9 submitted by National Research Centre on Plant Biotechnology and 38S102 submitted by ICAR-Indian Agricultural Research Institute, New Delhi) were used directly and their sequence data have been deposited in the GenBank nucleotide database^2^ with the accession number ANHQ00000000, AJIL00000000, AORR00000000, AORQ00000000, AEEW00000000, AORS00000000, AORT00000000, LACS00000000, LACT00000000, LACU00000000, and MKXH00000000, respectively. BioEdit was used to conduct the local blast to identify the corresponding sequences (*E*-value, 1.0; Matrix, BLOSSUM 62). Editseq from Lasergene 7 was used to translate all gene sequences to amino acid sequences. The software DNAMAN 6.0 was then used to create a multiple sequence alignment. Once one different amino acid appeared, the amino acid substitution was counted in the multiple alignment.

### Total RNA Extraction and Transcription Level Analysis

Total RNA from wheat at 12, 18, 24, 36, 48, 72, 120, and 168 hpi and RNA from urediniospores and germ tubes of CYR32 were extracted using the TRIzol^TM^ reagent (Invitrogen, Carlsbad, CA, United States) according to the manufacturer’s instructions. Potential contaminating DNA was digested with *DNase* I (Promega, Madison, WI, United States). The quality of RNA was evaluated by electrophoresis on ethidium bromide-stained 1.0% agarose gels. First-strand cDNA was synthesized using the Go Script Reverse Transcription System (Promega). Oligo (dT)18 was used as a primer, and the reverse transcription reaction was incubated according to the Go Script Reverse Transcription System protocol. After a 1:10 dilution, 2 μL of the synthesized cDNA was used for qRT-PCR.

The transcription levels of *Pst_8713* in different treatments were measured using qRT-PCR following the procedure described by Wang et al. (2012). Housekeeping gene elongation factor 1 (EF1) from *Pst* was used as an endogenous reference to normalize gene expression across different *Pst* samples (Ling et al., 2007). The transcription level of *Pst_8713* was calculated using the 2^-ΔΔCT^ method (Livak and Schmittgen, 2012). Transcript abundance was assessed with three independent biological replicates.

### Validation of the Pst_8713 Signal Peptide

The validation of predicted Pst_8713 signal peptides was conducted using the yeast secretion system described by Jacobs et al. (1997). pSUC2T7M13ORI (pSUC2) carries a truncated invertase, SUC2, which lacks both initiation methionine and signal peptide, was used. The recombinant plasmid pSUC2-Pst_8713 and the control plasmids pSUC2-Avr1b and pSUC2-Mg87 were transformed into yeast strain YTK12 (Gietz et al., 1995), respectively. All transformants were cultured on the yeast minimal medium with sucrose (CMD-W medium: 0.67% yeast N base without amino acids, 0.075% tryptophan dropout supplement, 2% sucrose, 0.1% glucose, and 2% agar) and only YTK12 strain transformed with an pSUC2 vector could grow on the CMD-W medium. To test whether Pst_8713-SP was functional by yeast growth assays, single colonies of YTK12 with or without expressing various plasmids were cultured in the YPRAA medium containing raffinose as the carbohydrate source (1% yeast extract, 2% peptone, 2% raffinose, and 2 mg/mL antimycin A). The untransformed YTK12 strain, YTK12 strain transformed with an empty pSUC2 vector and the first 25 amino acids of Mg87 protein from *M. oryzae* were used as negative controls. YTK12 strain carrying the signal peptide of Avr1b was used as a positive control. This assay ensured three biological repeats.

### Transient *in planta* Expression Assays for Subcellular Localization

For subcellular localization in wheat mesophyll protoplasts, the method of wheat mesophyll protoplast isolation, PEG-calcium transfection of plasmid DNA, and protoplast culturing, we referred to the protocol used for *Arabidopsis* mesophyll protoplasts (Yoo et al., 2007). For subcellular localization in *N. benthamiana*, *an A. tumefaciens* strain GV3101 containing the binary vector expression constructs was cultured on the LB medium supplemented with 50 μg/mL kanamycin, 50 μg/mL gentamycin, and 50 μg/mL rifampicin to the late log phase. The cells were collected and resuspended in an infiltration medium (10 mM MgCl_2_, 10 mM MES, and 200 mM acetosyringone, pH 5.6) to an OD600 of 0.8. The suspensions of *A. tumefaciens* were infiltrated into tobacco leaves of 6 weeks old *N. benthamiana* plants. Plant tissue samples were harvested from infiltrated tobacco leaves at 2 days after infiltration. Epidermal tissues of tobacco leaves were tore down for microscopical observation with an Olympus BX-51 microscope (Olympus Corporation, Japan). The 488-laser line with appropriate emission filter was used to image GFP autofluorescence.

### Western Blotting Analysis

The total proteins in wheat mesophyll protoplasts or infiltrated tobacco leaves were extracted using the method described by Moffett et al. (2002). The extracted protein solution (16 μL) was loaded on a 15% SDS-PAGE gel. Proteins were transferred to a microporous polyvinylidene fluoride (PVDF) membrane (Millipore, Billerica, MA, United States), and incubated in the blotting buffer (5% non-fat milk powder in TBS). Proteins were detected using mouse-derived GFP antibodies (Sungene, Tianjing, China) incubated overnight at 4°C. Membranes were washed and incubated with horseradish peroxidase-conjugated anti-mouse secondary antibody (Sungene, Tianjing, China) and chemiluminescence substrate for detection (Sigma, Tokyo, Japan).

### *A. tumefaciens* Infiltration Assays for Suppression of BAX/INF1 Induced Programmed Cell Death (PCD)

Constructs were introduced to *A. tumefaciens* strain GV3101 by electroporation (Hellens et al., 2000; Wise et al., 2006). Positive transformants were selected with kanamycin (50 μg/mL) and rifampicin (50 μg/mL). Individual colonies were verified by PCR amplification using the vector primers. For infiltration into leaves, recombinant strains of *A. tumefaciens* were harvested and resuspended in 10 mM MgCl_2_ to a final OD600 of 0.2, then incubated at room temperature in dark for 2 h prior to infiltration. To analyze Pst_8713 suppression of BAX/INF1 induced cell death, *A. tumefaciens* cell suspension carrying Pst_8713 was infiltrated initially, and *A. tumefaciens* cells carrying BAX/INF1 were infiltrated into the same site 24 h later. The symptoms were monitored 3–5 days after inoculation with BAX/INF1 and photos were taken. The leaves were decolorized using ethanol. *A. tumefaciens* cells carrying eGFP or Avr1b were infiltrated and used as negative and positive controls, respectively. Each assay was consisted of at least three leaves on three plants and ensured three biological repeats.

### Bacterial TTSS-Mediated Overexpression in *N. benthamiana* Plants

The pEDV6:Pst_8713 and pEDV6:dsRED (control) constructs were introduced into the *P. fluorescens* strain EtHAn by electroporation. Cells of the *P. fluorescens* strain EtHAn were diluted to an OD600 of 0.6 and infiltrated into 6 weeks old leaves of *N. benthamiana*. The leaf samples harvested at 24 hpi were stained with aniline blue (Hood and Shew, 1996) to observe callose deposition. Specimens were examined with an Olympus BX-51 microscope (Olympus Corporation, Japan). Bacterial growth levels in tobacco were measured by cutting tissues around the infiltrated point with amounts to 50 mg fresh weight. Tissues were homogenized in 200 μL of the inoculation buffer. The bacterial suspensions were diluted and plated on KB solid medium with the appropriate antibiotics. The total RNA and cDNA of all samples at 24 hpi were obtained using the procedural methods described above. Subsequently, SYBR green qRT-PCR assays were conducted to measure the transcription levels of three *N. benthamiana* defense-related genes (*PR1a*, *PR1b*, and *WRKY12*). The *N. benthamiana* housekeeping gene *NbActin* was used as the endogenous reference to normalize the gene expression and the 2^-ΔΔCT^ method was used to measure the relative gene expression levels (Livak and Schmittgen, 2012). Experiments were repeated three times.

### Bacterial TTSS-Mediated Overexpression in Wheat Plants

The pEDV6:Pst_8713 construct was introduced into the *P. fluorescens* strain EtHAn by electroporation. For transient expression in *N. benthamiana* cells, bacterial cells carrying Pst_8713 were cultured overnight in the KB medium with appropriate antibiotics at 28°C (Yin and Hulbert, 2011), then collected and resuspended in an infiltration medium (10 mM MgCl_2_). The *P. fluorescens* strain EtHAn was diluted to an OD600 of 0.6 and infiltrated into the second leaves of wheat cultivar AvS using a syringe without a needle. The infiltrated wheat plants were maintained in a growth chamber at 25°C. The wild type strain EtHAn was used as a PTI trigger in wheat AvS. Wheat leaves infiltrated with recombinant EtHAn carrying the red fluorescent protein (RFP, dsRED) or the bacteria avirulent protein AvrRpt2 were used as negative or positive control, respectively. At 48 hpi, phenotypes were examined and samples were harvested. To examine the suppression of callose deposition, leaf samples were stained with aniline blue as described previously (Hood and Shew, 1996). Specimens were examined using an Olympus BX-51 microscope (Olympus Corporation, Japan). All data were compared to that of wheat leaves infiltrated with the EtHAn strain carrying dsRED. Experiments were repeated three times. The method of measuring bacterial growth levels *in planta* was descripted previously.

In the ETI suppression assays, the second leaves of wheat cultivar Suwon11 were first infiltrated with recombinant EtHAn suspension cells using the method described previously. Twenty-four hours later, the infiltrated leaves were inoculated with the isolate of avirulent *Pst* race CYR23 at the infiltration sites. The inoculated leaves were then sampled 24 h post-inoculation ((hpi)) for histochemical observation. Hydrogen peroxide accumulation was detected using DAB coloration (Thordal-Christensen et al., 1997). Wheat leaves were immersed immediately in a diaminobenzidine (DAB) solution. A total of 0.1 g of DAB powder (Sigma-Aldrich, St. Louis, MO, United States) was dissolved in 100 mL of water and adjusted to a pH of 3.8 by adding HCl, then stored for 8 h under light at 25°C. To visualize the necrotic cell death, leaf segments were fixed and decolorized in ethanol/acetic acid (1:1 v/v). The autofluorescence of the attacked mesophyll cells was observed under a fluorescence microscope (excitation filter 485 nm, dichromic mirror 510 nm, barrier filter 520 nm), and measured using DP-BSW software to determine the necrotic cell area. The disease phenotype was observed constantly 7 days post-treatment and photos were taken 10 days later. All data were compared to that of wheat leaves infiltrated with the EtHAn strain carrying dsRED. Experiments were repeated three times.

### BSMV-Mediated *Pst_8713* Gene Silencing

BSMV-HIGS was carried out as previously described by Wang et al. (2012). To silence *Pst_8713*, two BSMV constructs (BSMV:Ta-Pst_8713-1as and BSMV:Pst_8713-2as) were used to inoculate wheat seedlings. BSMV:TaPDS containing the wheat phytoene desaturase (PDS) gene and BSMV:00 were used as controls for the BSMV infection test. Mock inoculations were performed with 1× FES buffer as described (Wang et al., 2012). The fourth leaves were further inoculated with fresh CYR32 urediniospores at 9 days after virus inoculation and the wheat plants were maintained at 16°C ± 2°C (plants were placed in a dark chamber for 24 h and then relocated to a growth chamber under normal growth conditions). The phenotypes of the fourth leaves were observed and photographed at 14 days after *Pst* inoculation and the leaves were sampled for RNA extraction and genomic DNA isolation. qRT-PCR was used to evaluate the silencing efficiencies of *Pst_8713* as described above. To measure the *Pst* biomass in the infected wheat leaves, DNA quantification of the single-copy target genes *PsEF1* (from *Pst*) and *TaEF1* (from wheat) was carried out using qRT-PCR as previously described (Panwar et al., 2013; Liu J. et al., 2016). Total genomic DNA of Suwon11 or *Pst* isolate CYR32 was used to prepare standard curves which derived from seven serial dilutions for each, and the correlation coefficients for the analysis of the dilution curves were above 0.99. The relative quantities of the PCR products of the *Pst* gene *PsEF1* and the wheat gene *TaEF1* in infected wheat leaves were calculated using the standard curves to quantify *Pst* and wheat genomic DNA, respectively. Cytological analyses were performed to characterize *Pst* growth and the host response in wheat plants. Decolorized leaf segments were stained with wheat germ agglutinin (WGA) conjugated to Alexa-488 (Invitrogen, Carlsbad, CA, United States) (Ayliffe et al., 2011). Hydrogen peroxide accumulation was detected as described for DAB staining. The stained samples were examined under an Olympus BX-51 microscope (Olympus Corporation, Japan). Three biological replicates were used in this assay.

## Results

### Identification of the Candidate Effector Pst_8713

**FIGURE 1:**
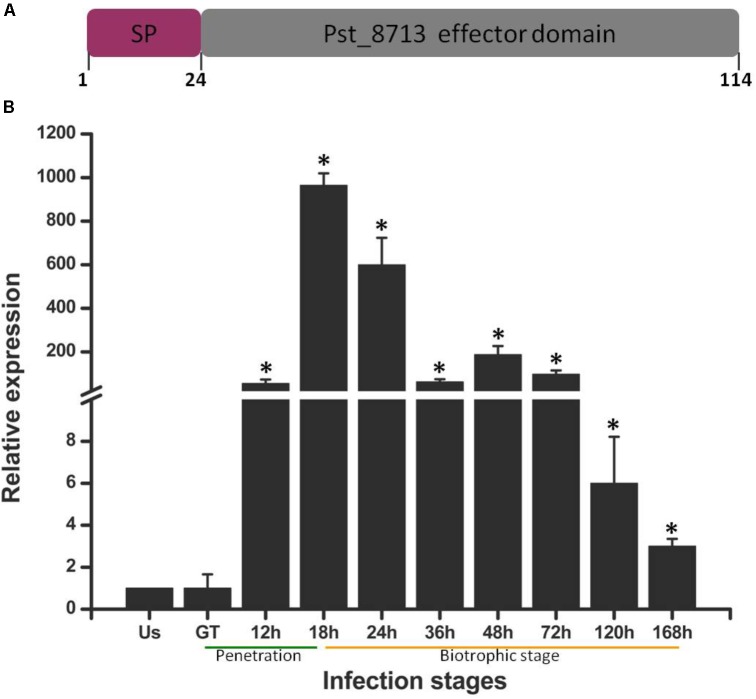
*Pst_8713* is significantly up-regulated during *Pst*-infection of wheat. **(A)** The primary structure of Pst_8713. SP, signal peptide. **(B)** Transcript levels of Pst_8713 at various stages of *Pst* infection. US, Urediniospores; GT, Germinated tube of US, 12–168 h, wheat leaves infected with *Pst* at 18–168 hpi. The relative gene quantification was calculated by the comparative Ct method with *Pst* endogenous gene *EF1* as an internal standard and was relative to that of US. Bars indicate means of three independent biological replicates (±SE). Asterisks indicate significant differences (*P* < 0.01) relative to the US sample determined using Student’s *t*-test.

In our previous study, a number of *Pst* secreted proteins were identified (Zheng et al., 2013). To further decipher possible roles of effectors during *Pst* infecting wheat, we screened several candidate secreted proteins using the criteria below: with unknown function, rich in cysteine residue (≥4), highly expressed in haustoria, ≤300 amino acids in length (Saunders et al., 2012). Of which, we selected one highly up-regulated gene *Pst_8713* for further study. The transcript levels of *Pst_8713* was measured at different infection stages, including non-germinated urediniospores, germ-tubes, and infected wheat tissues harvested from 12 to 168 hpi (**Figure 1B**). The expression of *Pst_8713* was highly up-regulated in infected wheat leaves (from 12 to 168 hpi) compared to the non-germinated urediniospores and germ tubes, and the expression level reached its peak at 18 hpi when *Pst* haustoria were formed in wheat. Our results showed that the transcript level of *Pst_8713* is highly elevated during haustoria formation and continued rising throughout the infection phase.

The Pst_8713 protein is small in size with 114 amino acids and the first 24 aa of N-terminal is a predicted signal peptide (**Figure 1A**), which is a key feature for secretion. Annotation analysis revealed that Pst_8713 is a hypothetical protein, and shared the same homology with 100% identity with the hypothetical protein PSTG_01796 (GenBank Accession: KNF05168). Sequence information of Pst_8713 has been submitted to the Genbank (Accession: MG674423).

### *Pst_8713* Is Unique to *Pst* and Shows a Low Level of Intraspecific Polymorphism

Many effectors are reported to be unique to specific pathogens, and exhibit species specific virulence (Stergiopoulos and de Wit, 2009; Giraldo and Valent, 2013). BLAST analysis revealed that *Pst_8713* has no homologues in any other published genome sequences. To determine if *Pst_8713* is polymorphic among different *Pst* isolates, we compared its amino acid sequences with 11 isolates from China, the US, the UK, and India. The sequences are identical among the isolates, except that isolate PST-130 from the US has two amino acids change (**Supplementary Figure S1**). These data indicated that Pst_8713 is a *Pst*-specific candidate effector with a very low level of polymorphism within *Pst*.

### Secretion Validation of the N-Terminal Signal Peptide of Pst_8713

**FIGURE 2:**
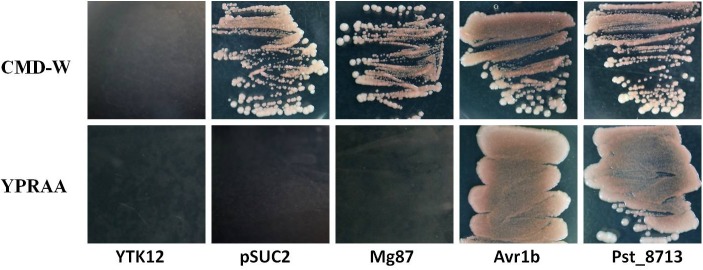
Functional validation of the signal peptide of Pst_8713. The sequence of the putative Pst_8713 signal peptide was fused in-frame to the invertase sequence in the pSUC2 vector and transformed into yeast strain YTK12. The untransformed YTK12 strain, empty pSUC2 vector and the first 25 amino acids of non-secreted Mg87 protein from *Magnaporthe oryzae* were used as negative controls, and the oomycete effector Avr1b from *Phytophthora sojae* was used as a positive control. Only yeast strains that are able to secrete invertase can grow on YPRAA media.

To test the secretion function of the N-terminal signal peptide of Pst_8713, we adopted the yeast signal peptide screen trap (YSST), a genetic assay which based on the requirement of invertase secretion for yeast growth on media with sucrose or raffinose as the sole carbon source (Jacobs et al., 1997; Oh et al., 2009; Tian et al., 2015). The predicted signal peptide sequence of Pst_8713 was fused to the mature sequence of yeast invertase in the vector pSUC2T7M13ORI (pSUC2) (Jacobs et al., 1997) and then transformed into the invertase secretion deficient strain YTK12 (Oh et al., 2009). For negative controls, we tested the empty pSUC2 vector and the pSUC2 fused with the first 25 amino acids of *Magnaporthe oryzae* protein Mg87 which was not predicted to be secreted (Gu et al., 2011). The predicted signal peptide of effector Avr1b from *Phytophthora sojae*, reported as a secretory leader was used as a positive control (Gu et al., 2011). Similar to Avr1b, the Pst_8713-fused construct enabled the invertase mutant yeast strain to grow on CMD-W medium (yeast growth without invertase secretion) and YPRAA medium (yeast growth only when invertase is secreted) (**Figure 2**). By contrast, when the construct fused with Mg87 N-terminus, the transformed yeast strains did not grow on YPRAA medium (**Figure 2**). This result verified the secretion function of the putative N-terminal signal peptide of Pst_8713.

### Pst_8713 Is Localized to the Plant Cytoplasm and Nucleus

**FIGURE 3:**
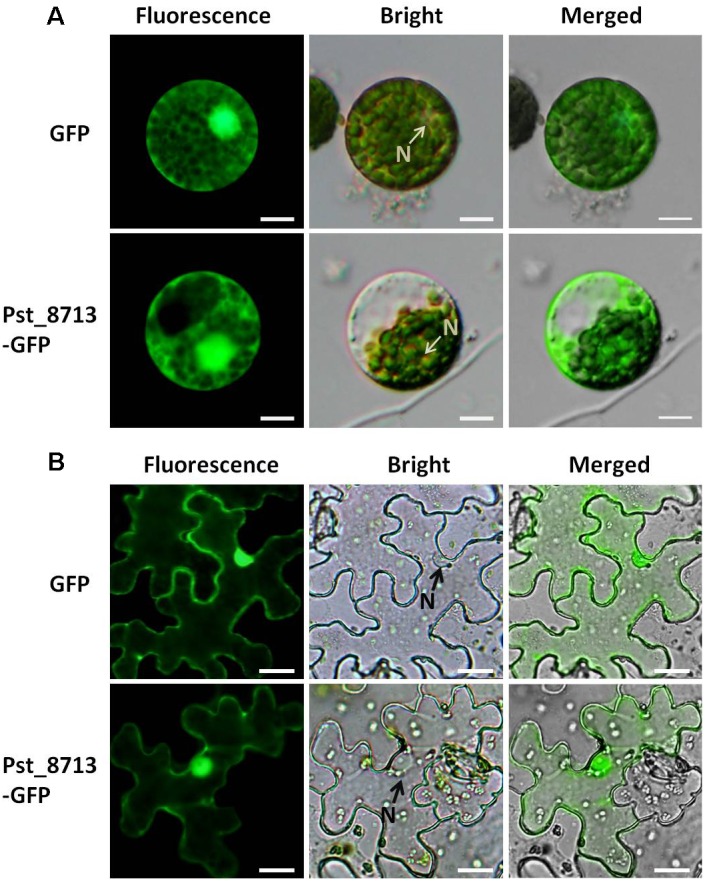
Localization of Pst_8713 in cytoplasm and nucleus. **(A)** GFP and Pst_8713-GFP fusion proteins in wheat mesophyll protoplasts with PEG-mediated transformation. N, nucleus. Bar = 50 μm. **(B)** Constructs of GFP and Pst_8713-GFP fusion proteins transformed into *Agrobacterium tumefaciens* and infiltrated into leaves of 6 weeks old *Nicotiana benthamiana.* N, nucleus. Bar = 10 μm.

It has been reported that effectors of filamentous fungal pathogens can be translocated into host cells to target diverse organelles (Rafiqi et al., 2012). To identify the subcellular localization of Pst_8713, transient expression analyses of the Pst_8713-GFP fusion protein in *N. benthamiana* leaves and wheat mesophyll protoplasts were conducted. The control expressing only GFP with the cauliflower mosaic virus 35S promoter exhibited fluorescence throughout the whole cell (cytoplasm and nucleus). The fluorescence of the Pst_8713-GFP fusion protein was also observed in cytoplasm and nucleus of *N. benthamiana* cells and wheat mesophyll protoplasts (**Figure 3**). The results of the Western blot analyses confirmed that the Pst_8713-GFP fusion protein was expressed in both *N. benthamiana* and wheat (**Supplementary Figure S2**).

### Pst_8713 Suppresses PCD Induced by Mouse Pro-apoptotic Protein-BAX and *Phytophthora infestans* PAMP-INF1 in *N. benthamiana*

**FIGURE 4:**
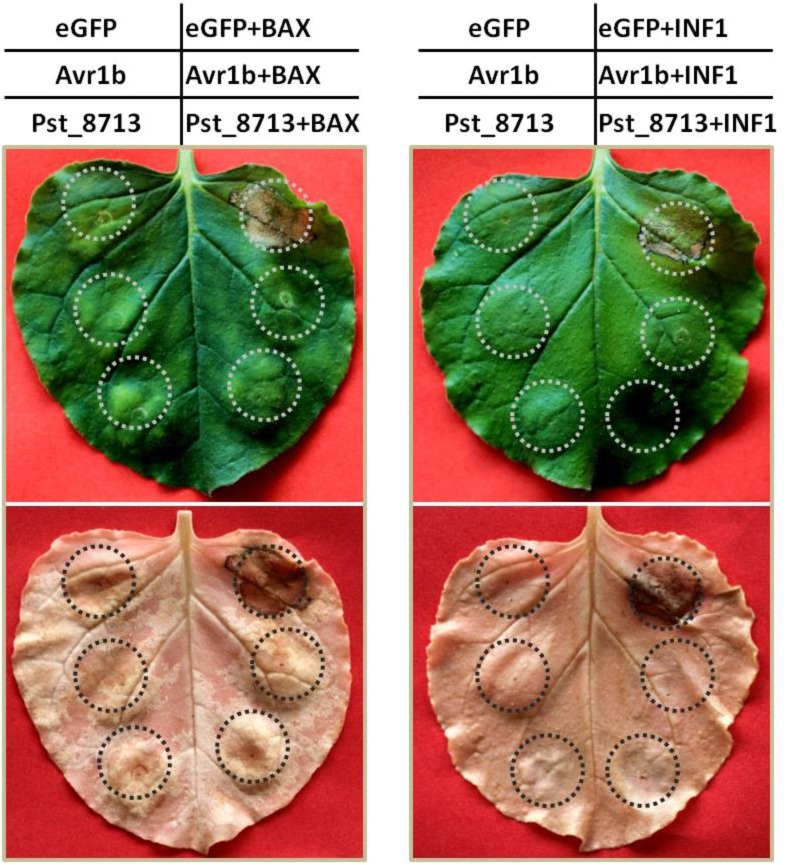
Overexpression of Pst_8713 in *N. benthamiana* suppressed programmed cell death triggered by BAX and PAMP-INF1. *N. benthamiana* leaves were infiltrated with *A. tumefaciens* cells containing PVX vector carrying eGFP (negative control), Avr1b (positive control) or Pst_8713, respectively, by inoculation with *A. tumefaciens* cells carrying PVX:BAX/INF1 24 h later. Photos were taken 5 days after infiltration, and the leaves were decolorized with ethanol.

We determined the function of Pst_8713 using an *A. tumefaciens*-mediated transient expression assay in *N. benthamiana*. BAX, a death-promoting protein of the Bcl-2 family in mouse proteins, was able to PCD when expressed in tobacco, similar to the plant defense related HR (Lacomme and Santa Cruz, 1999). We therefore determined the effector virulence function by detecting whether overexpression of Pst_8713 in the model plant *N. benthamiana* can suppress BAX-triggered PCD. The phenotype was observed 5 days post-BAX infiltration. The infiltration of the bacteria suspension carrying pGR107-eGFP did not result in PCD, whereas the sites infiltrated with pGR107-eGFP and then challenged with the *Agrobacterium* strain with pGR107-BAX 24 h later, exhibited pronounced cell death (**Figure 4**). By contrast, the sites infiltrated with pGR107-Avr1b and then challenged with BAX showed no PCD, indicating that PCD induced by BAX was successfully suppressed by Avr1b as reported by Dou et al. (2008). Similarly, the infiltration with pGR107-Pst_8713 successfully suppressed PCD caused by BAX (**Figure 4**). To further confirm the suppression effect of Pst_8713 to PCD, we also tested the effect of Pst_8713 on the suppression of PCD induced by INF1, a PAMP from *P. infestans* (Kamoun et al., 1997; Catanzariti et al., 2006). The transient expression of INF1 in tobacco leaves through *Agrobacterium* infiltration triggered significant PCD, and PCD was inhibited by Avr1b, but not by eGFP (**Figure 4**). Similar to Avr1b, Pst_8713 suppressed PCD induced by INF1 when infiltrated 24 h prior to INF1 incubation. These results indicated that overexpression of Pst_8713 suppresses PCD induced by BAX and PAMP-INF1, respectively, and alludes to the virulence function of Pst_8713.

### Delivering Pst_8713 in Wheat Suppresses PTI-Related Callose Deposition

**FIGURE 5:**
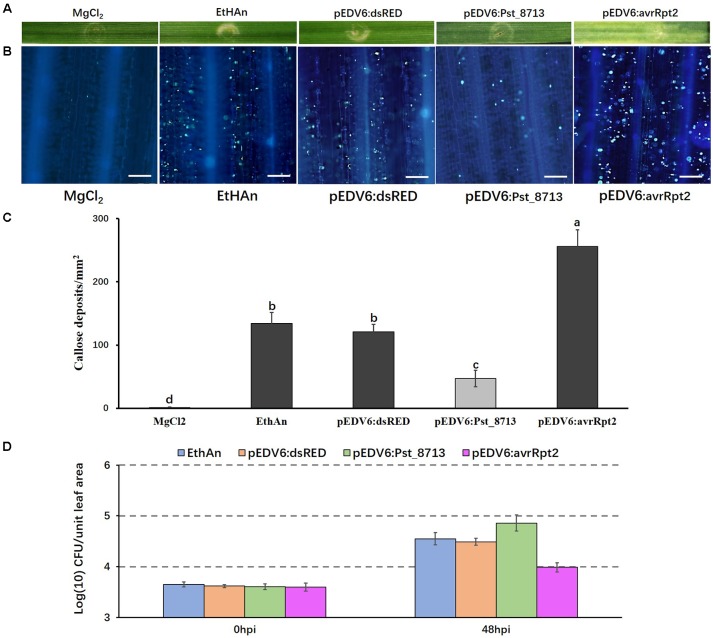
Overexpression of Pst_8713 in wheat (AvS) suppressed PTI-related callose deposition. **(A)** Phenotypes of the wheat leaves infiltrated with the MgCl_2_ buffer (MOCK), EtHAn, pEDV6:dsRED, pEDV6:Pst_8713, and pEDV6:AvrRpt2at 72 h after infiltration. **(B)** The wheat leaves inoculated with MgCl_2_ buffer, EtHAn, pEDV6:dsRED, pEDV6:Pst_8713, or pEDV6:AvrRpt2 were examined for callose deposition by epifluorescence microscopy after aniline blue staining. Bar = 100 μm. **(C)** Average number of callose deposits/mm^2^ in wheat leaves inoculated with MgCl_2_ buffer, EtHAn, pEDV6:dsRED, pEDV6:Pst_8713, or pEDV6:AvrRpt2. Bars indicate means ± SE of three independent biological replicates with 30 unit areas per replicate. The different letters indicate significant differences (*P* < 0.05) according to analysis of variance (ANOVA). **(D)** Bacterial growth in EtHAn, pEDV6:dsRED, pEDV6:Pst_8713, or pEDV6:AvrRpt2 infiltrated wheat leaves. Numbers of bacteria were evaluated at 0 and 48 hpi. Each time-point is average of three biological replicates. Bacteria in wheat inoculating with EthAn carrying pEDV6:Pst_8713 grew ∼2.75-fold better than EthAn carrying pEDV6:dsRED.

Because Pst_8713 suppresses PCD induced by BAX or INF1, we decided to further investigate its function in suppressing host immunity. It has been reported that the effector detector vector (EDV) can deliver individual non-bacterial effectors to host plant cells using bacterial TTSS (Sohn et al., 2007). Pst_8713 was cloned into the expression/delivery vector pEDV6, and then delivered into wheat cv. AvS by the modified *P. fluorescens* strain EtHAn, which carries a functional TTSS (Thomas et al., 2009; Yin and Hulbert, 2011). EtHAn is non-pathogenic on wheat (Yin and Hulbert, 2011) and inoculation of EtHAn in wheat could not trigger a chlorotic or necrotic reaction phenotype on wheat plants (**Figure 5A**). However, callose deposition was observed on wheat leaves after inoculating with EtHAn (**Figure 5B**), indicating that infection with non-pathogenic EtHAn triggered PTI in wheat. EtHAn strain-carrying dsRED and *P. fluorescens* effector AvrRpt2 were used as negative and positive controls, respectively (Bent et al., 1994; Mindrinos et al., 1994; Yin and Hulbert, 2011). Wheat leaves delivered with avrRpt2 exhibited a noticeable chlorotic phenotype (**Figure 5A**), and had a significant level of callose accumulation through the infiltrated regions (**Figure 5B**). We did not see obvious chlorotic or necrotic reactions in pEDV6:dsRED- and pEDV6:Pst_8713-inoculated wheat plants, but a decreased callose deposition in pEDV6:Pst_8713-inoculated wheat plants was observed (**Figures 5A,B**). We measured the callose deposition in the infiltrated wheat leaves and the callose deposition of Pst_8713-infiltrated leaves decreased by 61% compared to that of wheat leaves introduced with the EtHAn strain carrying pEDV6:dsRED (**Figure 5C**). Since overexpressing Pst_8713 suppressed PTI, we further tested whether Pst_8713 could enhance growth of EthAn in wheat. As shown in **Figure 5D**. EthAn carrying pEDV6:Pst_8713 grew ∼2.75-fold better than EthAn carrying pEDV6:dsRED. Thus, the results suggested Pst_8713 delivered by EtHAn effectively suppresses PTI-associated callose deposition and promotes bacterial growth in wheat.

### Pst_8713 Reduces Expression of PTI-Associated Marker Genes in *N. benthamiana*

**FIGURE 6:**
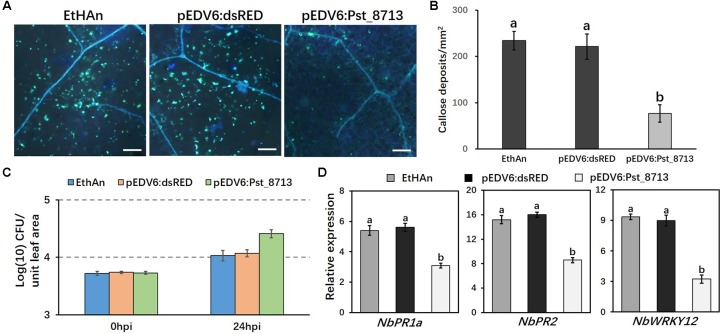
Overexpression of Pst_8713 in *N. benthamiana* suppressed expression of PTI-related marker genes. **(A)** Callose deposition in leaves of *N. benthamiana* inoculated with EtHAn, pEDV6:dsRED, and pEDV6:Pst_8713 stained with aniline blue. Bar = 200 μm. **(B)** The average number of callose deposits in tobacco leaves expressed with pEDV6:dsRED or pEDV6:Pst_8713. Bars indicate means ± SE of three independent biological replicates with 30 unit areas per replicate. The different letters indicate significant differences (*P* < 0.05) according to ANOVA. **(C)** Bacterial growth in EtHAn, pEDV6:dsRED, or pEDV6:Pst_8713 infiltrated tobacco leaves. Numbers of bacteria were evaluated at 0 and 24 hpi. Each time-point is average of three biological replicates. Bacteria in tobacco inoculating EthAn carrying pEDV6:Pst_8713 grew ∼2.2-fold better than EthAn carrying pEDV6:dsRED at 24 hpi. **(D)** Transcription level fold changes of plant defense-related genes *PR1a*, *PR2*, and *WRKY12* in leaves of *N. benthamiana* transformed with pEDV6: dsRED or pEDV6: Pst_8713 at 24 hpi with EtHAn. *NbActin* was used as the internal reference gene. Bars indicate means of three independent biological replicates (±SE) and different letters indicate significant differences (*P* < 0.05) according to ANOVA.

The experiment was further conducted in *N. benthamiana* instead of wheat to assess the expression of PTI-associated marker genes as reported by Qi et al. (2016). The pEDV6:dsRED (control) and pEDV6:Pst_8713 constructs were transferred to the EtHAn strain, which were then infiltrated into *N. benthamiana* leaves. We observed 65% lower level of callose deposition in Pst_8713-infiltrated leaves compared to the tobacco leaves with the EtHAn strain carrying pEDV6:dsRED (**Figures 6A,B**), indicating PTI suppression existed in Pst_8713-infiltrated tobacco leaves. The bacterial growth in *N. benthamiana* was also measured and EthAn carrying pEDV6:Pst_8713 grew nearly ∼2.2-fold better than EthAn carrying pEDV6:dsRED at 24 hpi (**Figure 6C**). To test whether Pst_8713 can decrease mRNA accumulation of tobacco immune marker genes, qRT-PCR was conducted to evaluate the expression of three genes which were confirmed to be related to *N. benthamiana* immune responses: pathogenesis-related gene *PR1a* and *PR2* (Sels et al., 2008) and the *WRKY12* transcription factor (Wang and Dixon, 2010; Kim et al., 2014). Expression levels of *PR1a*, *PR2*, and *WRKY12* were ∼1.8, 1.9, and 2.7-fold lower reduced in the Pst_8713-delivered plants (**Figure 6D**). These results indicated that Pst_8713 is able to suppress callose deposition, contributes to bacterial growth and suppresses expression of PTI-associated marker genes in *N. benthamiana*.

### Overexpression of Pst_8713 Weakens ETI in Wheat

**FIGURE 7:**
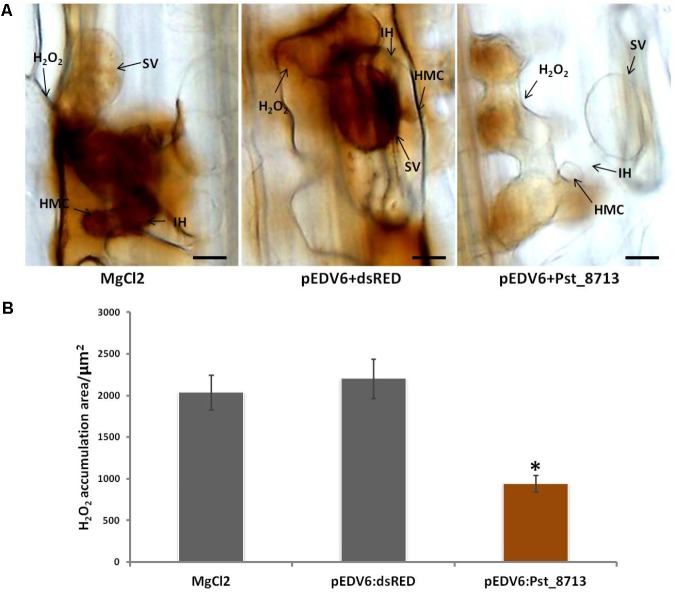
Overexpression of Pst_8713 suppressed ETI associated ROS in wheat Suwon11. **(A)** H_2_O_2_ accumulation at 24 hpi in wheat leaves infiltrated with MgCl_2_ buffer, pEDV6:dsRED, or pEDV6:Pst_8713, and 24 h later inoculated with the avirulent *Pst* isolate CYR23. After being stained with DAB, the samples were viewed under differential interference contrast optics. Bar = 10 μm. **(B)** Statistics of H_2_O_2_ accumulation area/μm^2^ in wheat leaves inoculated with CYR23 24 hpi after infiltrated with MgCl_2_ buffer, pEDV6:dsRED, or pEDV6:Pst_8713. Bars indicate means ± SE of three independent biological replicates with 30 infection sites per replicate. Asterisks indicate significant differences (*P* < 0.05) relative to the pEDV6:dsRED sample using Student’s *t*-test.

During the co-evolution of a pathogen and its host, some pathogen effectors may develop to be capable of suppressing plant ETI (Jones and Dangl, 2006). During the development of defense responses, pathogen recognition by plant cells leads to the rapid production of high levels of ROS (Wang et al., 2007), which is known as an important biomarker for plant ETI (Jones and Dangl, 2006; Wang et al., 2007). To test whether Pst_8713 is such an effector that can suppress host ETI, we inoculated *Pst* avirulent isolate CYR23 to leaves of wheat Suwon11 infiltrated with Pst_8713. Pst_8713 delivered by EtHAn did not cause noticeable necrotic or chlorotic reactions on Suwon11 plants which proved Suwon11 is a suitable wheat line to examine the virulence functions of *Pst* effectors. We took samples at 24 hpi for histological observation, when ROS principally produced in infection sites. Pst_8713 significantly suppressed ROS accumulation by 57%, compared to the control (**Figure 7**). Despite this, it was observed that the CYR23-infected wheat leaves after infiltration with Pst_8713 had no significant difference in disease phenotype, while the histological observation of HR, another biomarker for plant ETI (Wang et al., 2007) and DP-BSW software measurement indicated that Pst_8713 significantly decreased the necrotic cell death areas by 27.4% per infection site at 24 hpi (Supplementary Figure S3). These results indicated that Pst_8713 delivered by EtHAn weakens ETI in wheat.

### Transient Silencing of *Pst_8713* Decreases the Virulence of *Pst*

**FIGURE 8:**
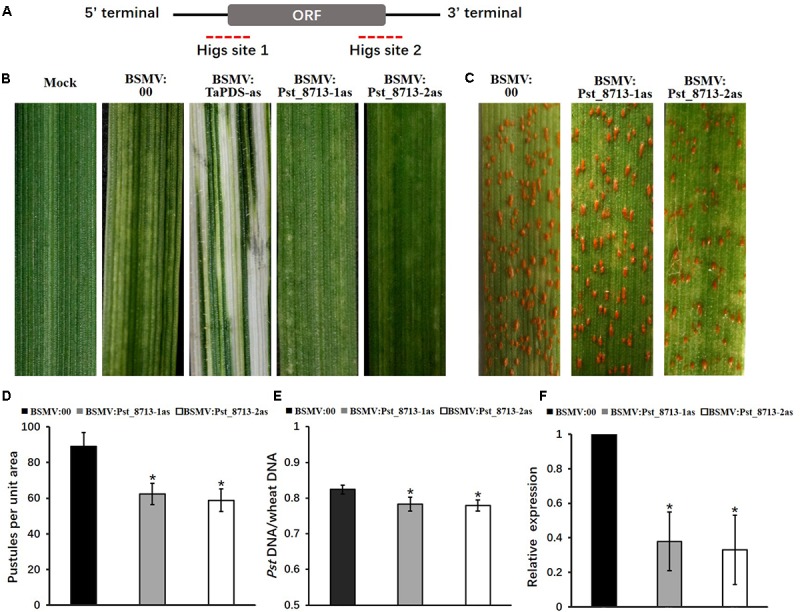
BSMV-mediated HIGS of *Pst_8713* decreased susceptibility of wheat plants to *Puccinia striiformis* f. sp. *tritici* (*Pst*). **(A)** *Pst_8713* transcript with CDS and 5′ and 3′UTRs. The dotted parts are two specific sequence regions for HIGS. **(B)** Phenotypes of the fourth leaves of wheat plants after inoculation with FES buffer (mock), BSMV:00 (control), BSMV:TaPDS, BSMV:Pst_8713-1as, and BSMV:Pst_8713-2as. **(C)** Phenotypes of the wheat leaves pre-inoculated with BSMV:00 (control), BSMV:Pst_8713-1as, and BSMV:Pst_8713-2as and then inoculated with *Pst* virulent isolate CYR32. **(D)** Quantification of *Pst* uredinia formation. The means and standard errors were from three biological replicates, and for each replicate, at least six treated leaves were used for uredinia accumulation. **(E)** Fungal biomass measurements using qRT-PCR analysis of total genomic DNA extracted from control (BSMV:00-inoculated) and *Pst_8713*-silenced wheat plants. Ratio of total *Pst* genomic DNA to total wheat genomic DNA was assessed using the *Pst* gene *PsEF1* and the wheat gene *TaEF1*. The means and standard errors were from three biological replicates. **(F)** The relative expression of *Pst_8713* after knocking down. The expression values are relative to the *Pst* endogenous gene *EF1*, with the empty vector (BSMV:00) set at 1. In **(D–F)**, samples were taken at 14 day post-inoculation with *Pst*. Asterisks indicate significant differences (*P* < 0.05) using Student’s *t*-test.

In absence of a stable genetic transformation system, HIGS mediated by BSMV was used to knock-down the expression of *Pst_8713* (Nowara et al., 2010; Yin and Hulbert, 2015; Cheng et al., 2017). Primers were designed to amplify two fragments across the coding region to specifically silence *Pst_8713* (**Figure 8A**). All BSMV-inoculated plants of Suwon11 displayed chlorotic stripes and mosaic symptoms at 9 days post-inoculation (dpi). In plants with the silenced wheat phytoene desaturase *TaPDS* gene, a bleaching phenotype was observed at 9 dpi, suggesting that the BSMV-HIGS system was functionally effective (**Figure 8B**). The fourth leaves of wheat plants were then inoculated with *Pst* virulent isolate CYR32, and photographed at 12 dpi. The phenotypes showed that BSMV:Pst_8713-1as and BSMV:Pst_8713-2as had reduced sporulation compared to BSMV:00 (control) leaves (**Figure 8C**). The number of uredinia of BSMV:Pst_8713-1as and BSMV:Pst_8713-2as were reduced by 30 and 34%, respectively (**Figure 8D**). In addition, qRT-PCR analysis showed that fungal biomass was also significantly decreased in the *Pst_8713*-silenced wheat plants compared to the control wheat plants (**Figure 8E**). The *Pst_8713* transcripts levels of the two fragments were effectively reduced by 62.1 and 67%, respectively (**Figure 8F**). Our results showed that silencing of Pst_8713 alters the *Pst* virulence phenotype which illustrates the virulence function of Pst_8713.

### Silencing of *Pst_8713* Impairs *Pst* Haustoria Formation and Marginally Increases H_2_O_2_ Accumulation During the Wheat–*Pst* Compatible Interaction

**FIGURE 9:**
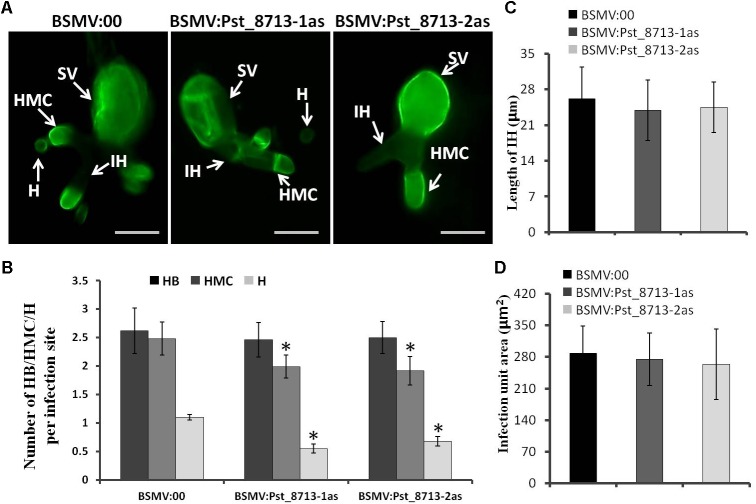
Histological observation of fungal growth in wheat plants inoculated first with BSMV:00 or recombinant BSMV and then inoculated with isolate CYR32 **(A)** Fungal growth at 18 h after CYR32 inoculation. The fungal structure was stained with wheat germ agglutinin (WGA) and observed under a fluorescence microscope. Bars = 20 μm. **(B)** The average number of HB, HMC and H in HIGS plants infected by CYR32. **(C)** Hyphal length, which is the mean distance from the junction of the sub-stomatal vesicle of the hypha to the tip of the hypha, measured using DP-BSW software. **(D)** Infection area, the mean area of the expanding hyphae plus the host cells, was calculated using DP-BSW software (unit in ×10^3^/μm^2^). All bars indicated means ± SE of three independent biological replicates with 50 unit areas per replicate, Asterisks indicate significant differences (*P* < 0.05) using Student’s *t*-test. SV, sub-stomatal vesicle; HMC, haustorial mother cell; IH, infection hypha, H, haustorium, and HB, hypha branch.

**FIGURE 10:**
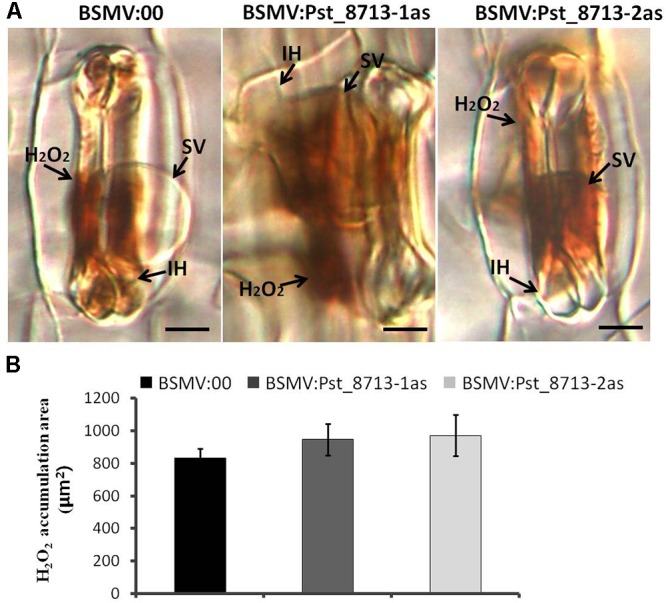
Histological observation of host response in wheat plants inoculated first with BSMV:00 or recombinant BSMV, then with isolate CYR32. **(A)** H_2_O_2_ accumulation in BSMV silenced leaves at 18 h after *Pst* inoculation. The fungal structure was stained with DAB and observed under differential interference contrast optics. Bars = 10 μm. **(B)** Statistics of H_2_O_2_ accumulation. The H_2_O_2_ accumulation area were calculated using DP-BSW software (unit in ×10^3^/μm^2^). All bars indicated means ± SE of three independent biological replicates with 50 unit areas per replicate.

Based on the phenotype variation of wheat leaves inoculated with BSMV:Pst_8713-1as and BSMV:Pst_8713-2as, we assessed the development of *Pst* and host responses in the HIGS plants inoculated with *Pst.* At 18 hpi, the number of haustorial mother cells (HMC) and haustoria (H) were significantly decreased (**Figures 9A,B**). There were no significant difference in the number of hyphal branches (HB), the length of infection hypha (IH), and infection area in the wheat seedlings inoculated with BSMV:Pst_8713-1as and BSMV:Pst_8713-2as compared to the control (**Figures 9A–D**). DAB staining showed that the H_2_O_2_ accumulation in wheat mesophyll cells inoculated with BSMV:Pst_8713-1as and BSMV:Pst_8713-2as at 18 hpi were increased compared to the control (**Figure 10A**), while the statistics of H_2_O_2_ accumulation implied that this increase was not significant (**Figure 10B**). In conclusion, Pst_8713 impairs the formation of *Pst* haustoria and affects the host immunity response.

## Discussion

Pathogen effectors are very important molecules that interfere with host immunity (Giraldo and Valent, 2013), and many effectors from diverse pathogens can enhance pathogenicity and subvert plant defense (Speth et al., 2007). In this study, we demonstrate that Pst_8713 is involved in *Pst* pathogenesis by reducing plant immunity.

The Agrobacterium-mediated transient expression approach enables high-throughput functional analysis of effectors (Jamir et al., 2004; Li et al., 2009; Wang et al., 2011). Using this approach, we determined that *Pst_8713* in *N. benthamiana* suppresses BAX- and INF1-triggered PCD. Because INF1 is a PAMP from *P. infestans* and can trigger PTI-related PCD in *N. benthamiana* (Kamoun et al., 1997), our results suggested that Pst_8713 is able to suppress PTI-associated PCD. In addition, we applied an EDV in the EtHAn strain to express and deliver Pst_8713 to plant cells. Overexpression of Pst_8713 in wheat leaves led to the inhibition of PTI-related callose deposition. Furthermore, Pst_8713 overexpression in tobacco leaves affected the expression levels of the defense marker genes *PR1a, PR2*, and *WRKY12* in response to PTI cues. Previous studies reported that most effectors of the oomycete *Hyaloperonospora arabidopsidis* and bacterial *PtoDC3000* can suppress PTI in plants elicited by non-pathogenic microorganisms and pathogen PAMPs (Guo et al., 2009; Thomas et al., 2009; Fabro et al., 2011). Liu C. et al. (2016) reported one *Pst* effector, PEC6, suppressed PTI responses and targeted adenosine kinases to favor fungal growth. Several pathogen effectors have been proven to suppress ETI triggered by overexpressing single *Avr* genes in plants carrying corresponding R genes (Jones and Dangl, 2006), but few direct evidence demonstrate effectors suppress ETI responses in the incompatible interaction between host plants and avirulent pathogens. Our findings showed that Pst_8713 could significantly decrease ROS bursts and HR in avirulent isolate CYR23 infected-wheat plants which are two hallmarks for plant ETI (Hemetsberger et al., 2012). It was indicated that the host ETI was weakened, though there is no significant difference in disease phenotype. Meanwhile, the results suggested that Pst_8713 is a minor effector for *Pst* pathogenicity. It is reported that *Xanthomonas campestris* pv. *vesicatoria* type III effectors can also suppress HopA1-induced ETI (Fujikawa et al., 2006). Guo et al. (2009) tested 35 DC3000 type III effectors for their ability to suppress pHIR11-dependent ETI responses and found that the majority of the effectors were capable of suppressing ETI. Our data showed that Pst_8713 not only suppresses PTI but also ETI responses, indicating its multilayered functions in *Pst* virulence. We speculated that Pst_8713 may possibly possess multiple activities to target simultaneously target different components of PTI and ETI.

Using BSMV-mediated HIGS, a useful tool to study genes of obligate biotrophic pathogens, we silenced the *Pst_8713* gene. The decreased number of pustules and haustoria implied Pst_8713’s function in promoting virulence, even though the infection hyphal length and infection area did not significantly change. ROS, an important marker of host response, was measured and the H_2_O_2_ area was marginally decreased, which indicated Pst_8713 plays a partial role in inverting host immunity. We speculated that knockdown of *Pst_8713* resulted in less obvious phenotypes might be due to the function redundancy of effectors, as reported in the *Melampsora lini* effector repertoire (Lawrence et al., 2010) and the *P. syringae* pv. *tomato* DC3000 type III secretion effectors (Kvitko et al., 2009). Despite this issue, the results confirmed the role of Pst_8713 in contributing to *Pst* pathogenicity.

The molecular mechanisms of how Pst_8713 suppresses host defenses and contributes to the pathogenicity of *Pst* are compelling and will be a major focus of our next study. Rust candidate effectors were reported to target multiple plant departments and plant protein complexes (Lorrain et al., 2018), so future work should entail seeking the possible targets of Pst_8713 in its host which can help to explore molecular basis of defense suppression and its possible role in pathogenesis.

## Author Contributions

MZ, XW, and ZK contributed to the design of the work. MZ, SJ, ZC, JX, and SC performed the experiments. MZ, JW, and CT analyzed the sequencing data. MZ and JW wrote the manuscript and CT revised the manuscript. XW and ZK were responsible for all aspects of this study.

## Conflict of Interest Statement

The authors declare that the research was conducted in the absence of any commercial or financial relationships that could be construed as a potential conflict of interest.

## Footnotes

1 http://www.cbs.dtu.dk/services/SignalP/

2 http://www.ncbi.nlm.nih.gov/genome/genomes/2580?

## Supplementary Material

FIGURE S1 Amino acid sequences alignment of Pst_8713 among 11 *Puccinia striiformis* f. sp. *tritici* (*Pst*) isolates including 1 Chinese isolate (CYR32), 3 US isolates (PST-21, PST-43, PST-78, and PST-130), 2 UK isolates (08/21 and 87/7), and 4 Indian isolates (Race-31, Race-K, Race-Yr9, and 38S102). The Pst_8713 protein only had 2 amino acid substitutions in PST-130.

FIGURE S2 Western blot of Pst_8713 in wheat and tobacco plants. **(A)** GFP and GFP-Pst_8713 fusion proteins in wheat mesophyll protoplasts with the GFP-antibody. **(B)** GFP and GFP-Pst_8713 fusion proteins in *Nicotiana benthamiana* with the GFP-antibody

FIGURE S3 Overexpression of Pst_8713 suppressed ETI associated HR in wheat Suwon11. Statistics of necrotic area/μm^2^ in wheat leaves inoculated with CYR23 24 hpi after infiltrated with MgCl_2_ buffer, pEDV6:dsRED or pEDV6:Pst_8713. The means and standard errors were from three biological replicates. The asterisk indicates significant difference (*P* < 0.05) relative to the pEDV6:dsRED sample.

TABLE S1 Primers used in this study.
