# Supplementary figures and images for: Candidate Effector Pst_8713 Impairs the Plant Immunity and Contributes to Virulence of Puccinia striiformis f. sp. tritici
Source: Front Plant Sci. 2018 Sep 11;9:1294. doi: 10.3389/fpls.2018.01294 (PMC6141802; doi:10.3389/fpls.2018.01294)

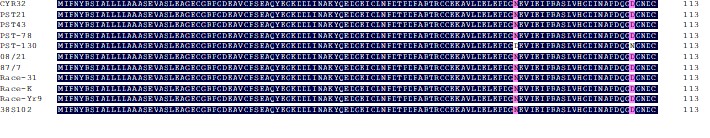

Supplement: FIGURE S1 — Amino acid sequences alignment of Pst_8713 among 11 Puccinia striiformis f. sp. tritici (Pst) isolates including 1 Chinese isolate (CYR32), 3 US isolates (PST-21, PST-43, PST-78, and PST-130), 2 UK isolates (08/21 and 87/7), and 4 Indian isolates (Race-31, Race-K, Race-Yr9, and 38S102). The Pst_8713 protein only had 2 amino acid substitutions in PST-130. [file Image_1.JPEG]

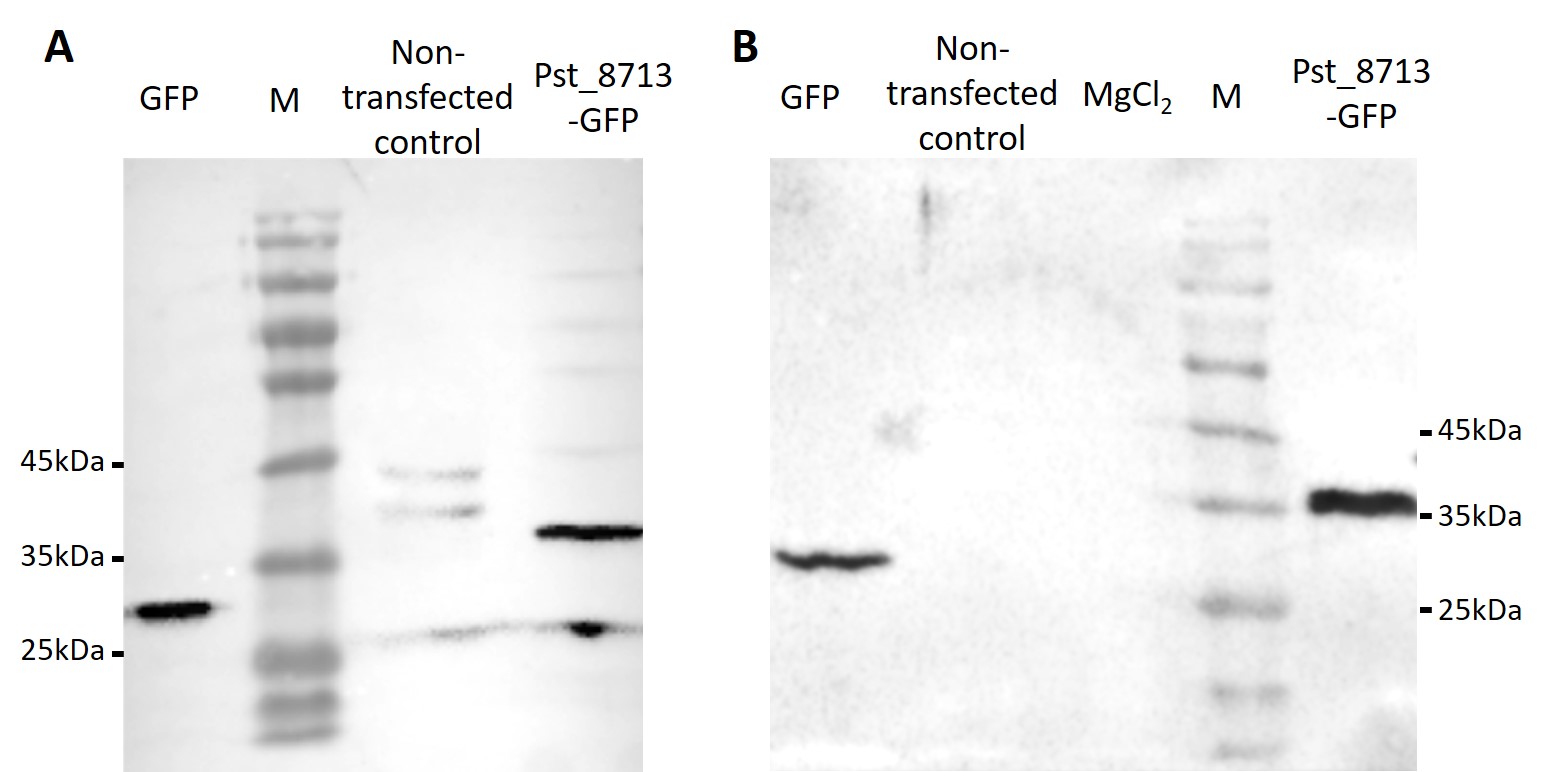

Supplement: FIGURE S2 — Western blot of Pst_8713 in wheat and tobacco plants. (A) GFP and GFP-Pst_8713 fusion proteins in wheat mesophyll protoplasts with the GFP-antibody. (B) GFP and GFP-Pst_8713 fusion proteins in Nicotiana benthamiana with the GFP-antibody [file Image_2.JPEG]

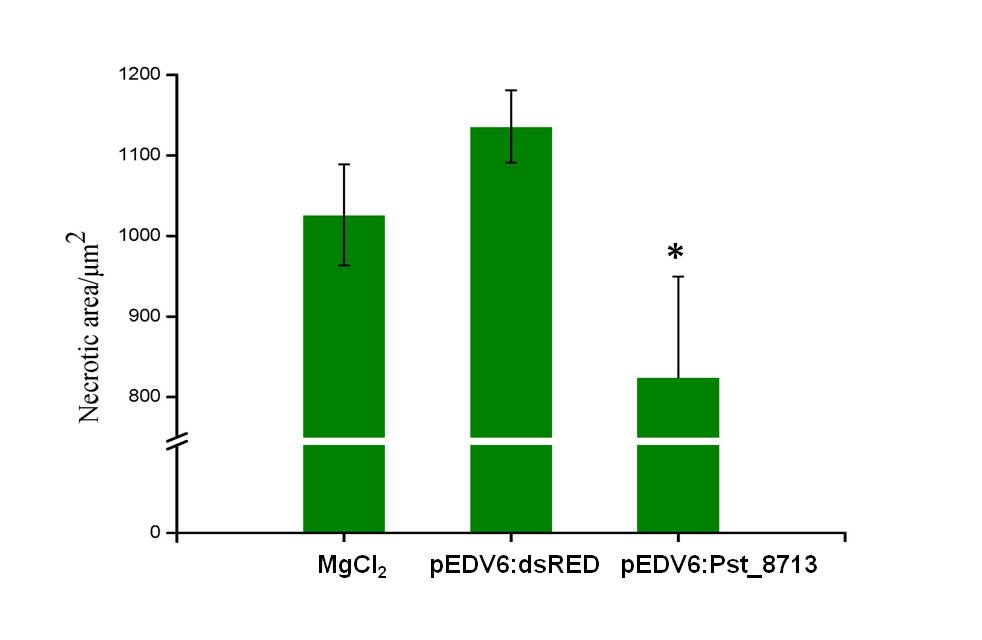

Supplement: FIGURE S3 — Overexpression of Pst_8713 suppressed ETI associated HR in wheat Suwon11. Statistics of necrotic area/μm2 in wheat leaves inoculated with CYR23 24 hpi after infiltrated with MgCl2 buffer, pEDV6:dsRED or pEDV6:Pst_8713. The means and standard errors were from three biological replicates. The asterisk indicates significant difference (P < 0.05) relative to the pEDV6:dsRED sample. [file Image_3.JPEG]
