# Supplementary material for: Candidate Effector Pst_8713 Impairs the Plant Immunity and Contributes to Virulence of Puccinia striiformis f. sp. tritici
Source: Front Plant Sci. 2018 Sep 11;9:1294. doi: 10.3389/fpls.2018.01294 (PMC6141802; doi:10.3389/fpls.2018.01294)
Supplement: TABLE S1 — Primers used in this study. [file Table_1.DOCX]

| **Primer name** | **Primer 5'-3'** | **Purpose** |
| --- | --- | --- |
| PsEF1-qRT-F | TTCGCCGTCCGTGATATGAGACAA | The endogenous reference for normalization in qRT-PCR |
| PsEF1-qRT-R | ATGCGTATCATGGTGGTGGAGTGA | The endogenous reference for normalization in qRT-PCR |
| TaEF-qRT-F | TGGTGTCATCAAGCCTGGTATGGT | Calculation of fungal biomass |
| TaEF-qRT-R | ACTCATGGTGCATCTCAACGGACT | Calculation of fungal biomass |
| Pst_8713-q-F | CTCAGAAGCACAATACAAGGG | qRT-PCR for validation of the expression patterns of Pst_8713 |
| Pst_8713-q-R | GTGCAAAGTCTGGGGTGTC | qRT-PCR for validation of the expression patterns of Pst_8713 |
| NbActin-F | GTTGCTATACAAGCTGTTCTCTCG | qRT-PCR for *NbActin* |
| NbActin-R | GTCAAGACGAAGAATGACATGTGG | qRT-PCR for *NbActin* |
| NbPR1a-F | CGACCAGGTAGCAGCCTATG | qRT-PCR for *NbPR1a* |
| NbPR1a-R | TCTCAACAGCCTTAGCAGCC | qRT-PCR for *NbPR1a* |
| NbPR2-F | GGGCTGTTAATTTGCAGTATCC | qRT-PCR for *NbPR2* |
| NbPR2-R | GGTTTATAACATCTTGGTCTGATGG | qRT-PCR for *NbPR2* |
| NbWRKY12-F | CTCATCAGCTAGTTCATTTGATGC | qRT-PCR for *NbWRKY12* |
| NbWRKY12-R | AGCTCGGTCTTTGTTCTAAAAGC | qRT-PCR for *NbWRKY12* |
| 8713-sp-EcoRI-F | GGAATTCATGATCTTCAATTACCGTTCAATC | Clone Pst_8713-sp to pSUC2 for secretion validation |
| 8713-sp-XhoI-R | CCGCTCGAGGGCCTTGAGGGATGCAAC | Clone Pst_8713-sp to pSUC2 for secretion validation |
| Pst_8713-PacI-F1 | CCTTAATTAACCCATCGACTCCCCCTAC | Clone Pst_8713-1as to the r vector for HIGS |
| Pst_8713-NotI-R1 | ATAAGAATGCGGCCGCGGGCCTTCCGCACTCTC | Clone Pst_8713-1as to the r vector for HIGS |
| Pst_8713-PacI-F2 | CCTTAATTAACTGTGTTAGACAAACTTAAACCTGA | Clone Pst_8713-2as to the r vector for HIGS |
| Pst_8713-NotI-R2 | ATAAGAATGCGGCCGCAGAAATACAAATCCAACATCTCACT | Clone Pst_8713-2as to the r vector for HIGS |
| Pst_8713-SmaI-F | GTACCCGGGATGATCTTCAATTACCGTTCAAT | Clone Pst_8713 to PVX for expression in *N. benthamiana* |
| Pst_8713-NotI-R | ATAAGAATGCGGCCGCTTAAATGCAGTCGTTTCCATC | Clone Pst_8713 to PVX for expression in *N. benthamiana* |
| eGFP-SmaI-F | GTACCCGGGATGGTGAGCAAGGGCGA | Clone eGFP to PVX for expression in *N. benthamiana* |
| eGFP-SalI-R | CATGTCGACTTACTTGTACAGCTCGTCCATG | Clone eGFP to PVX for expression in *N. benthamiana* |
| Avr1b-SmaI-F | GTACCCGGGATGCGTCTATCTTTTGTGCTT | Clone Avr1b to PVX for expression in *N. benthamiana* |
| Avr1b-NotI-R | ATAAGAATGCGGCCGCTCAGCTCTGATACAGGTGAAAG | Clone Avr1b to PVX for expression in *N. benthamiana* |
| BAX-SmaI-F | GTACCCGGGATGGACGGGTCCGGGGA | Clone BAX to PVX for expression in *N. benthamiana* |
| BAX-NotI-R | ATAAGAATGCGGCCGCGCCCATCTTCTTCCAGATGGTG | Clone BAX to PVX for expression in *N. benthamiana* |
| INF1-SmaI-F | GTACCCGGGATGAACTTTCGTGCTCTGTTCG | Clone INF1to PVX for expression in *N. benthamiana* |
| INF1-NotI-R | ATAAGAATGCGGCCGCTAGCGACGCACACGTAGACG | Clone INF1 to PVX for expression in *N. benthamiana* |
| Pst_8713-Gy-F | GGGGACAAGTTTGTACAAAAAAGCAGGCTTCGGAGAGTGCGGAAGGC | Clone Pst_8713 to pEDV6 for overexpression in wheat  and to pK7WGF2 for expression in *N. benthemiana* |
| Pst_8713-Gy-R | GGGGACCACTTTGTACAAGAAAGCTGGGTCTTAAATGCAGTCGTTTCCAT | Clone Pst_8713 to pEDV6 for overexpression in wheat  and to pK7WGF2 for expression in *N. benthemiana* |
| dsRED-Gy-F | GGGGACAAGTTTGTACAAAAAAGCAGGCTTCATGGCCTCCTCCGAGAAC | Clone dsRED to pEDV6 for overexpression in wheat |
| dsRED-Gy-R | GGGGACCACTTTGTACAAGAAAGCTGGGTCTTACAGGAACAGGTGGTGGC | Clone dsRED to pEDV6 for overexpression in wheat |
| Pst_8713- SalI-F | ACGCGTCGACATGGGAGAGTGCGGAAGGC | Clone Pst_8713 to pTF486 for expression in wheat protoplasts |
| Pst_8713-BamHI-R | CATGGGATCCGCAATGGTCATGATGATGATG | Clone Pst_8713 to pTF486 for expression in wheat protoplasts |
